# Research on Crack Propagation of Nitrate Ester Plasticized Polyether Propellant: Experiments and Simulation

**DOI:** 10.3390/ma17102180

**Published:** 2024-05-07

**Authors:** Hanwen Liu, Jiangning Wang, Xiaolong Fu

**Affiliations:** Xi’an Modern Chemistry Research Institute, Xi’an 710065, China; 3018001675@tju.edu.cn (H.L.); wangjiangning001@sohu.com (J.W.)

**Keywords:** NEPE propellant, crack propagation velocity, fracture toughness, stress intensity factor, bond-based peridynamic

## Abstract

To understand the fracture properties of the nitrate ester plasticized polyether (NEPE) propellant, single-edge notched tension (SENT) tests were carried out at room temperature (20 °C) under different tensile rates (10–500 mm/min). The mechanical response, crack morphology, evolution path, and crack propagation velocity during the fracture process were studied using a combination of a drawing machine and a high-speed camera. The mode I critical stress intensity factor *K_I_*_c_ was calculated to analyze the tensile fracture toughness of the NEPE propellant, and a criterion related to *K_I_*_c_ was proposed as a means of determining whether the solid rocket motors can normally work. The experimental results demonstrated that the NEPE propellant exhibited blunting fracture phenomena during crack propagation, resulting in fluctuating crack propagation velocity. The fracture toughness of the NEPE propellant exhibited clear rate dependence. When the tensile rate increased from 10 mm/min to 500 mm/min, the magnitude of the critical stress intensity factor increased by 62.3%. Moreover, numerical studies based on bond-based peridynamic (BBPD) were performed by modeling the fracture process of the NEPE propellant, including the crack propagation speed and the load–displacement curve of the NEPE propellant. The simulation results were then compared with the experiments.

## 1. Introduction

Solid propellants are the core of solid rocket motors (SRM). The nitrate ester plasticized polyether (NEPE) propellant has a much higher energy density than other propellants [1,2,3], and its mechanical, process, and safety properties are outstanding. Due to these excellent performances, the NEPE propellant has been widely used in SRM in recent years [4]. In terms of material composition, the NEPE propellant is a highly filled solid viscoelastic composite. It is comprised of poly (ether-urethane) as a binder, mixed nitric ester as a plasticizer, ammonium perchlorate (AP), aluminum particles (Al), and octogen (HMX) as solid fillers, and other additives. Throughout the entire life cycle of solid propellants, the processes of manufacturing, transportation, storage, and launch can potentially induce cracks. These damages will not only change the mechanical properties of propellant materials but also destroy the integrity of engine structures. Moreover, it will affect the combustion performance of solid propellants so that the SRM can not work normally, and even cause explosions or other accidents [5,6,7]. Therefore, it is essential to study the fracture properties of the NEPE propellant and establish a fracture model that can effectively predict the crack initiation and propagation.

The tensile fracture test can reveal the critical value of propellant fracture parameters, observe the crack propagation evolution, and analyze the stress–strain distribution and damage degree at the crack tip [8,9,10]. In a previous study, Wang et al. studied the impact of loading rates on the fracture properties of composite propellants [11]. The research demonstrated that the loading rate did not affect the shape and trend of the tensile fracture stress–strain curve of the propellant, and the tensile fracture toughness of the propellant could be improved by increasing the loading rate. Wang et al. prefabricated a crack for an AP/Al/CMDB propellant and carried out tensile tests, which can be used to study the fracture characteristics of cracked propellant grains in SRM and provide parametric support for the establishment of propellant fracture models and numerical simulations [12]. Wang et al. used self-developed in situ video imaging technology to characterize the crack propagation and crack path morphology of a hydroxyl-terminated polybutadiene (HTPB) propellant and studied the fracture characteristics of the HTPB propellant in detail [13].

In addition to tensile tests, many numerical methods have been developed to study the fracture behavior and crack propagation processes of composite materials [14,15]. Currently, the finite element method (FEM) is widely used to study the fracture characteristics of solid propellants, including the extended finite element method (XFEM) employing Cohesive Zone Models (CZMs) [16,17]. GAO B et al. used XFEM to study the crack propagation of the HTPB propellant in the 30% strain range [18]. The experimental results were in good agreement with the simulation results, demonstrating the feasibility of XFEM for simulating crack propagation in propellant materials. Cui et al. also took advantage of CZM in the context of modeling the behavior of propellants [19].

A competitive alternative to XFEM, especially for dynamic fracture and complex crack problems, is peridynamic (PD) developed by Silling [20,21,22]. PD is widely used to simulate the mechanical properties and damage behavior of various materials (metals, composites, etc.) including energetic materials [23,24,25,26,27]. DENG et al. modeled a polymeric-bonded explosive (PBX) and studied its dynamic damage response under impact load using bond-based peridynamic (BBPD) [28]. However, PD is far from sufficient in the field of energetic materials, especially for propellant materials, and there are almost no studies.

Currently, the fracture characteristics of NEPE propellants remain poorly understood, particularly with regard to the specific fracture process, which has a significant impact on the establishment of SRM integrity and safety evaluation criteria. Therefore, it is necessary to conduct further research into the fracture properties of NEPE propellants. Furthermore, the application of the material parameters obtained in the experiment to the simulation and establishment of the corresponding simulation model with appropriate numerical methods is also a problem worthy of further study.

In this work, the fracture characteristics of NEPE propellant were studied in detail. Based on a combination of a drawing machine and a high-speed camera, the crack morphology and evolution during the crack propagation were explored. The load–displacement curves and crack propagation velocity at different tensile rates were measured in experiments. Furthermore, the energy release rate and the stress intensity factor were calculated, and a criterion related to the stress intensity factor was proposed as a means of determining whether SRM can normally work. In addition, the BBPD was used to simulate and predict the crack propagation of the NEPE propellant for the first time.

## 2. Experiment Section

### 2.1. Material

The investigated NEPE propellant specimen was produced by Xi’an Modern Chemistry Research Institute (Xi’an, China), including polyether polyurethane as the polymer binder, AP, aluminum, and HMX as solid fillers, mixed nitric ester as the plasticizer, and other additives (combustion catalyst). The basic formulation of the NEPE propellant is denoted in Table 1.

### 2.2. Single-Edge Notched Tension Tests

**Figure 1:**
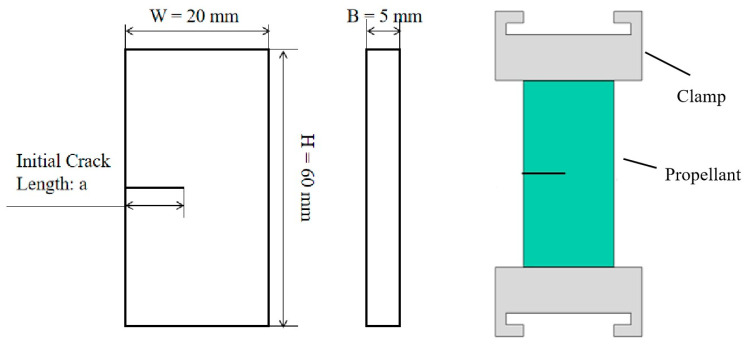
Dimensions of SENT test specimen.

The size of a SENT specimen is shown in Figure 1, with a width of W = 20 mm, a thickness of B = 5 mm and an effective length of H = 60 mm. The initial crack in the middle of the specimen was created by a ceramic knife, and the initial crack length was 10 mm. 

**Figure 2:**
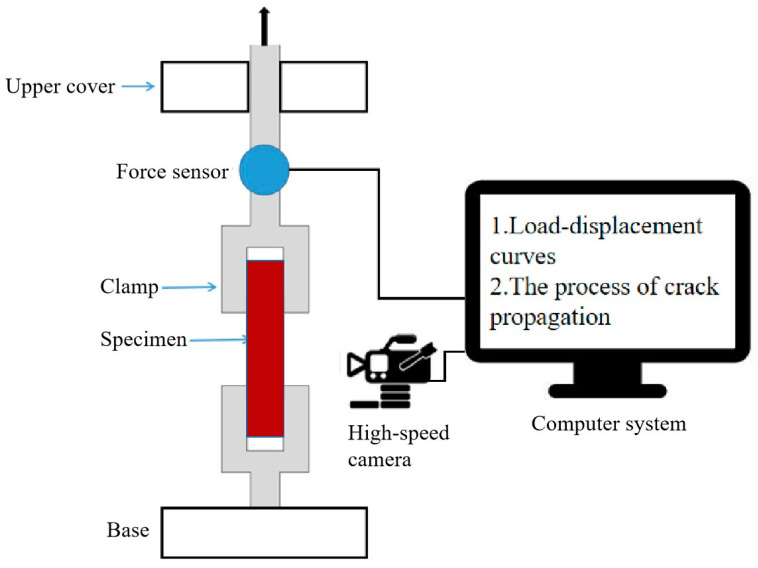
Schematic diagram of the experimental equipment.

In this paper, a uniaxial tensile machine (SAAS, Zhuhai, China) was equipped to carry out the single-edge notched tension tests. In order to obtain the crack-path morphology and cracking evolution of the NEPE propellant, a high-speed camera was positioned in close proximity to the tensile machine. The force sensor of the drawing machine has a maximum measuring range of 100 N and a measuring accuracy of 0.0001 N. The high-speed camera model is the “Revealer X190” (Fuhuang Junda High-Tech Information Technology Co, Hefei, China), with a maximum frame rate of 9300 fps and a full-frame resolution of 1280 × 1024 pixels. As shown in Figure 2, the tensile machine and high-speed camera are linked to the same computer, enabling the simultaneous triggering of both devices from the computer. 

The SENT tests were conducted with three different tensile rates: 10 mm/min, 100 mm/min, and 500 mm/min. Each set of experiments was repeated five times. Firstly, the propellant specimen and the fixture were bonded together with AB adhesive. Following the curing of the adhesive, the bonded assembly was installed on the stretching machine. Secondly, the high-speed camera was installed, and the appropriate frame rate was selected after focusing. When the tensile rate was 500 mm/min, the specimen fractured rapidly, and the frame rate of 1500 fps was selected by the high-speed camera. The frame rate selected for the other two tensile rates was 500 fps. Finally, the camera and the tensile machine commenced operation simultaneously. During the stretching process, the force sensor and computer system obtained the mechanical response of the specimen, while the camera recorded the process of crack propagation. 

## 3. Numerical Simulation Method

### 3.1. Liner Viscoelastic Constitutive Mode 

A solid propellant can be considered a linear viscoelastic material [13,29]. The objective of this study is to investigate the macroscopic fracture behavior of the propellant material, and the PD theory provides a damage criterion and function for describing the damage to materials. Therefore, the liner viscoelastic mode is employed for numerical simulation.

**Figure 3:**
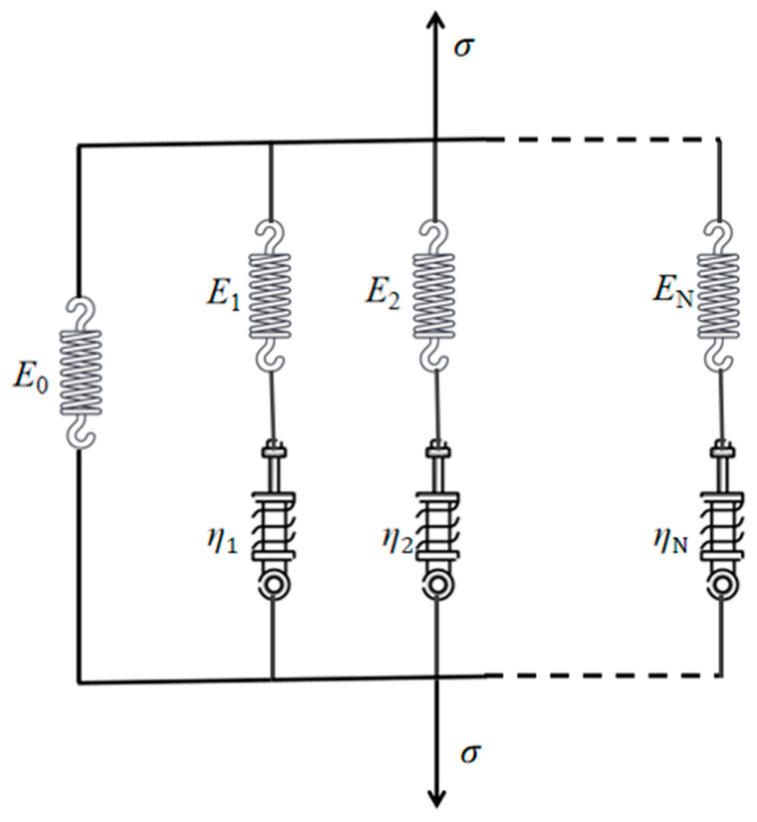
Generalized Maxwell model [30].

The mechanical properties of isotropic linear viscoelastic materials can be described by the generalized Maxwell model. As shown in Figure 3, this model consists of an elastic element (spring) and *n* components, which have both an elastic element and a viscous element (damper) in series. In the generalized Maxwell model, the stress relaxation modulus *E*(*t*) can be expressed with the Prony series as [30]

(1) $$E(t)=E_{\infty }+\sum_{n=1}^{N_{E}}\;E_{n}exp(-\frac{t}{\tau_{n}^{E}})$$

where $E_{\infty }$ is the long-term equilibrium modulus, $N_{E}$ stands for the number of terms of the Prony series, and *E_n_* and $\tau_{n}^{E}$ are model parameters of Prony series coefficients. *E*_0_ = $E_{\infty }+\sum_{n=1}^{N_{E}}\;E_{n}$ is the initial stress relaxation modulus. In numerical simulation, the relaxation modulus is selected in accordance with reference [31]. The parameters in the Prony series for the relaxation modulus of solid propellant are listed in Table 2.

### 3.2. Bond-Based Peridynamic

**Figure 4:**
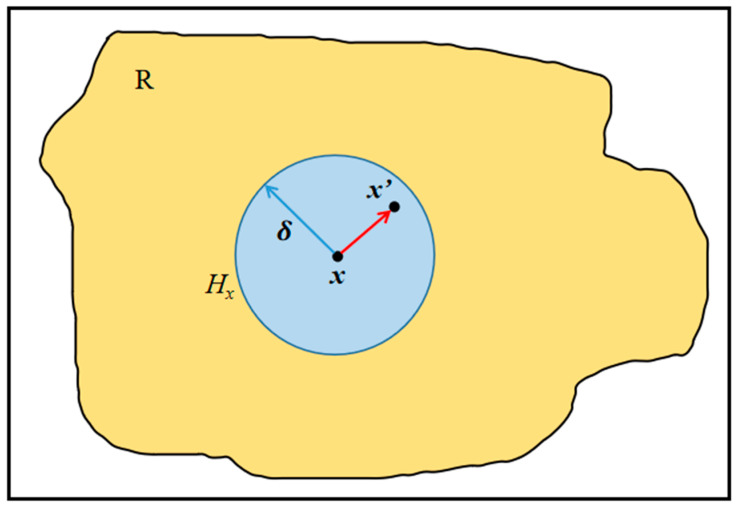
Material point *x* and the horizon *H_x_*.

The BBPD method was used in the numerical simulation. It assumes that in the material region *R*, each material point *x* can interact with other points *x*′ within a cut-off distance δ [32], also called horizon size, as seen in Figure 4, where *H_x_* denotes the horizon of *x*.

In peridynamics, the equation of motion can be expressed as [33]

(2) $$\rho \ddot{u}(x,t)=\int_{H}\;f\left(u(x,t),u^{\prime }(x,t),x,x^{\prime },t\right)dV_{x^{\prime }}+b(x,t)$$

where *ρ* is the mass density of the material point *x* and *u* stands for the displacement field. Superimposed ‘dots’ indicate material time derivatives while $dV_{x^{\prime }}$ represents the volume; *b* is the body force density.

**Figure 5:**
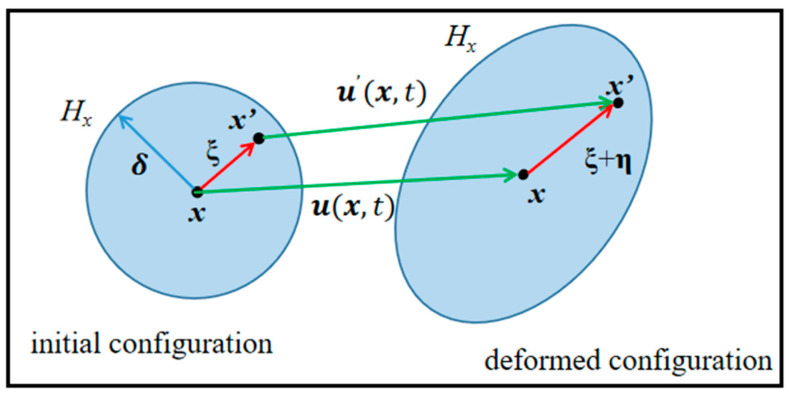
Initial and deformed configuration in PD.

The motion state of material point *x* depends only on its initial state and interaction with all material points *x*′ within the horizon. In the BBPD, the interaction between *x* and all other material points in the domain *H_x_* is called bond force f, which can be expressed as [34]

(3) $$f=cs\mu (t,\xi )\frac{∥\xi +\eta ∥}{\left(\xi +\eta \right)}$$

(4) $$s=\frac{∥\xi +\eta ∥-∥\xi ∥}{∥\xi ∥}$$

where *c* is the PD micro-modulus; μ and *s* represent the damage function and bond stretch, respectively. The bond force f is a function of the relative position vector ξ in the reference configuration and the relative displacement vector η in the current configuration [34]. As shown in Figure 5, they can be expressed as [35]

(5) ξ=x′−x

and

(6) $$\eta =u^{\prime }(x,t)-u(x,t)$$

The micro-modulus is a quantity that can be related to the material constant of classical continuum mechanics taking advantage of a quasi-continuum approach. For 2D plane stress, it can be written as $c=\frac{9E}{2\pi \delta^{3}}$, where *E* is the elasticity modulus of the materials.

In PD theory, when the elongation *s* between two material points exceeds its critical value *s*_c_, the bond will break; the critical stretch *s*_c_ depends on the critical energy release rate *G*_c_ of the material, and *s*_c_ can be expressed as $s_{c}=\sqrt{4\pi G_{c}/(9E\delta )}$ (in this study, *E* is the current Young’s modulus at time *t*). The force between two material points of matter in the equation of motion will disappear forever when damage occurs. In order to visualize the damage behavior of the material, the damage function μ is introduced [34]:

(7) $$\mu (t,\xi )=\left\{\begin{array}{c} 1,\mathrm{if}\;s<s_{c},0<t^{\prime }<t\\ 0,otherwise \end{array}\right.$$

The damage range of the material point *x* can be quantified by the damage variable φ defined as the ratio of the number of damage bonds at the material point to the initial total number of PD bonds [34]:

(8) $$\varphi (x,t)=1-\frac{\int_{H}\;\mu (t,\xi )dV^{\prime }}{\int_{H}\;dV^{\prime }}$$

**Figure 6:**
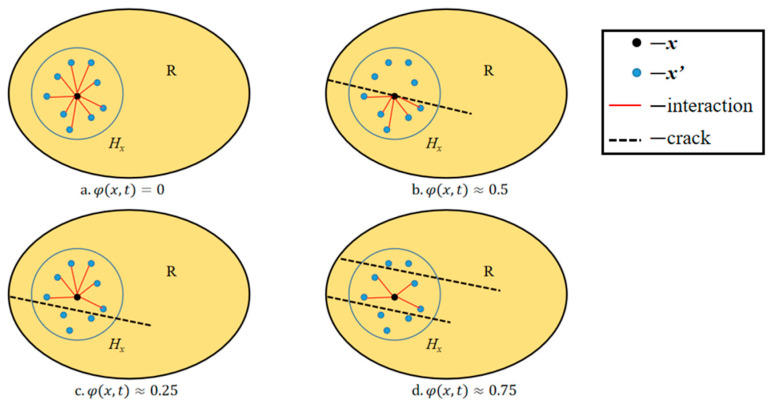
Effects of damage generation on interactions between material points.

The crack will be formed if the bonds between material points that appear continuous break. It indicates that there is no damage when the value φ equals 0. When the value φ reaches its maximum of 1, all bonds in the horizon are broken. The interaction of material points after crack formation is shown in Figure 6.

For the time integration, the explicit Velocity-Verlet algorithm is utilized [36,37]:

(9) $${\dot{u}}^{n+1/2}={\dot{u}}^{n}+\frac{\Delta t}{2}{\ddot{u}}^{n}$$

(10) $$u^{n+1}=u^{n}+\Delta t{\dot{u}}^{n+\frac{1}{2}}$$

(11) $${\dot{u}}^{n+1}={\dot{u}}^{n+1/2}$$

where Δt is the time step, which should not exceed the critical time step Δt_cri_ for stability reasons; Δt*_cri_* can be estimated as

(12) $$\Delta t_{cri}=\frac{\Delta x}{c_{k}}$$

where *c_k_* is the sound speed of the material while ∆x is the distance between two adjacent points. A time step of Δt = 0.8 Δt*_cri_* was employed in the simulation.

## 4. Result and Discussion

### 4.1. Experimental Results and Analysis

#### 4.1.1. Crack Morphology and Evolution Process

**Figure 7:**
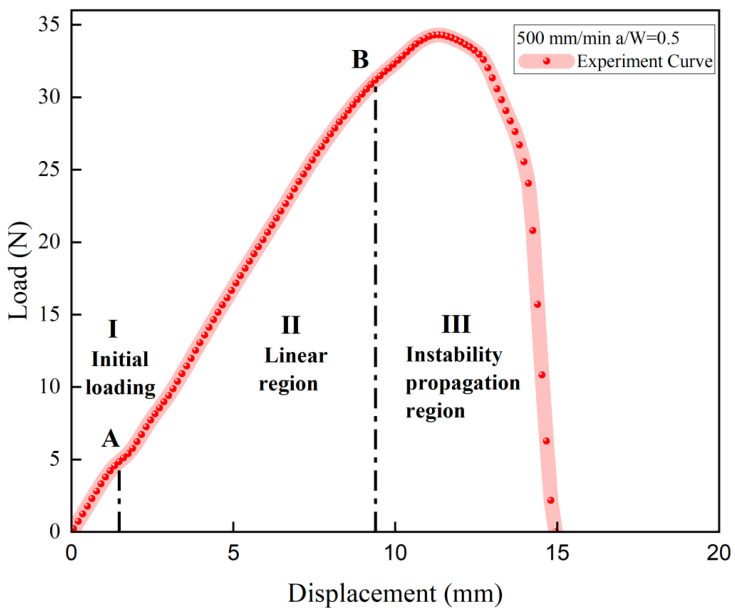
The typical Load–Displacement curve for a specimen.

**Figure 8:**
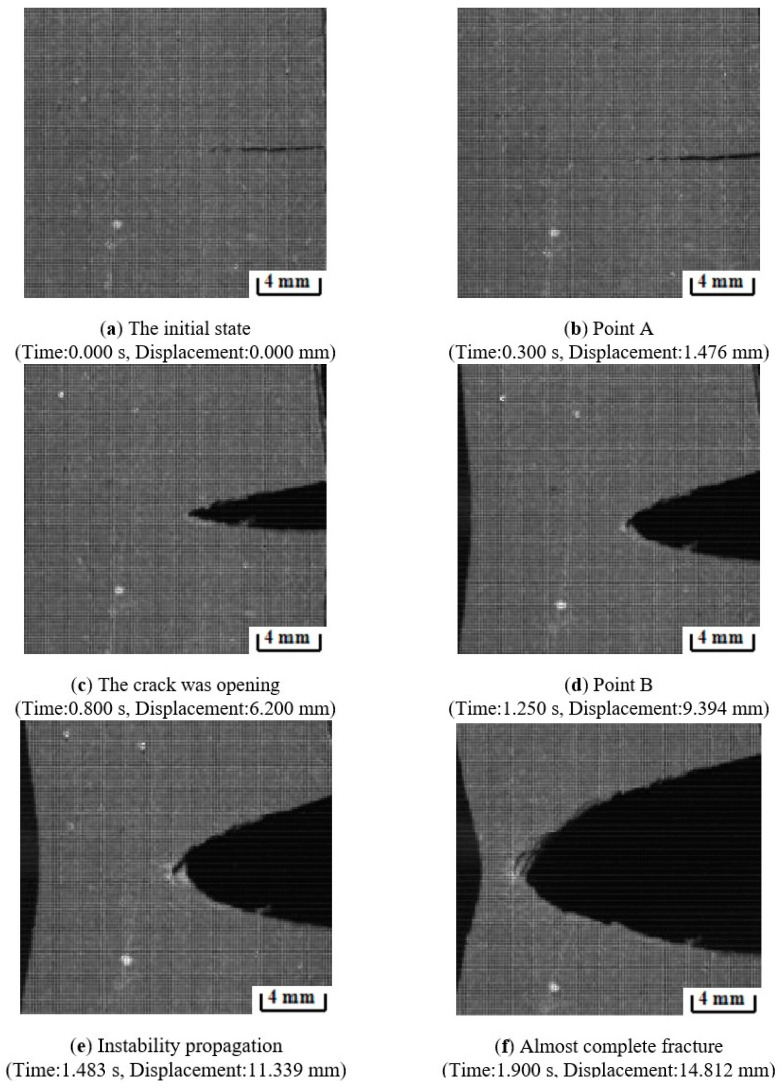
Tensile fracture process corresponding to Figure 7.

The experimental load–displacement curve can be found in Figure 7 and the associated crack morphology in Figure 8. The load–displacement curve is divided into three regions. Region I in Figure 7 (curve OA section) corresponds to Figure 8a,b. In Region I, the contact surface between the chuck of the stretching machine and the specimen is in the early warning state, and the contact effect has not reached the ideal state, so the experimental curves of the same group at this stage do not have strict parallel repeatability.

Once the chuck has achieved the desired contact effect with the test specimen, the crack propagation process enters its second stage (Region II in Figure 7 and Figure 8c,d). As shown in Figure 7 (curve AB section), the crack hardly spreads, and the load has a typical linear relationship with the displacement. In this region, the propellant material is in a linear viscoelastic state, and there is almost no damage inside the propellant during this stage. During the stretching process, the initial crack opens slowly until the crack opening displacement reaches a critical value (i.e., the microcrack initiation point). In fact, the crack can also expand at this stage, but the macrocrack propagation distance is extremely short and the propagation velocity is very slow as the microcracks continue to grow.

**Figure 9:**
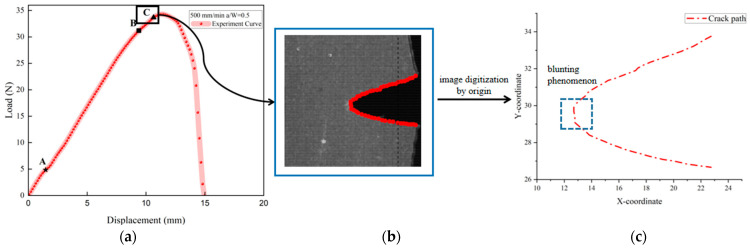
Image digitization processing of crack path: (**a**) load–displacement curve; (**b**) image digitization and crack feature point selection; (**c**) output the feature point coordinates in origin.

Then the tensile fracture enters the third stage (Region III) as shown in Figure 7 (curve BC section) and Figure 8e,f. In this region, the propellant is in the “damage evolution and crack propagation region”, and the growth trend of the characteristic curve starts to slow down near point B. This indicates that the elastic stiffness of NEPE propellant material begins to decrease. It is evident that obvious blunting occurs at the crack tip upon entering this region as shown in Figure 9. Then the load–displacement curve drops sharply, and during this process, the propellant crack exhibits obvious instability expansion until the specimen is completely fractured. The crack propagation velocity in this region increases exponentially, and multiple voids expand and merge simultaneously. The presence of a blunting fracture results in a non-monotonic increase in crack propagation velocity. The crack propagation velocity and crack tip characteristics have been analyzed in more detail in Section 4.1.2.

**Figure 10:**
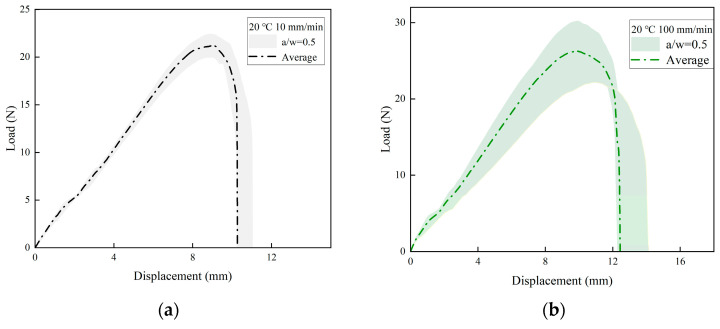

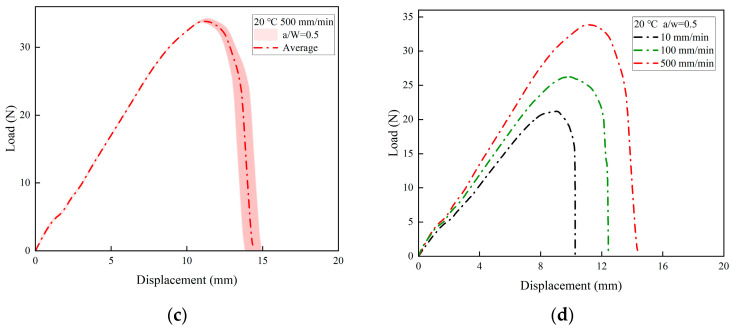
The relationship between load and displacement under different tensile rates: (**a**) 10 mm/min; (**b**) 100 mm/min; (**c**) 500 mm/min; (**d**) average load–displacement curves.

This study conducted SENT tests at three different tensile rates (10, 100, and 500 mm/min) with the same initial crack length (a/W = 0.5). The load–displacement curves of the specimens under the three tensile rates are presented in Figure 10a–c, respectively. Each curve represents the average value, while the shading behind each curve illustrates the error produced in repeated experiments. Figure 10d shows the average value of the load–displacement curves in the range of 10–500 mm/min tensile rates. In all cases, the characteristics of the load–displacement curves are found to be quite similar, which is consistent with the findings obtained using other propellant samples [9,13]. The results of the experiment indicate that the strength of the NEPE propellant increases as the tensile rate increases. The tensile fracture toughness of NEPE propellants exhibited a notable increase when the tensile rate was elevated from 10 mm/min to 500 mm/min.

#### 4.1.2. Determination Result and Analysis of Crack Tip Behavior

Wang’s study [38] indicates that blunting fracture is a phenomenon exclusive to the HTPB propellant, but the aforementioned study demonstrates that the NEPE propellant also exhibits an obvious blunting phenomenon during the crack propagation process. In previous studies, many scholars have monitored the fracture process using high-speed digital cameras and studied the crack characteristics with video imaging systems [9,12,39,40]. However, the video imaging system alone lacks accuracy, and thus one of the focuses of this study is to combine the video imaging system with image digitization technology in order to study the crack characteristics with the aim to improve the accuracy of the analysis.

The primary focus of the investigation was the behavior of the crack tip in region III of the displacement–load curve, and the magnification of the high-speed camera was increased to focus on the crack tip rather than the entire specimen. 

**Figure 11:**
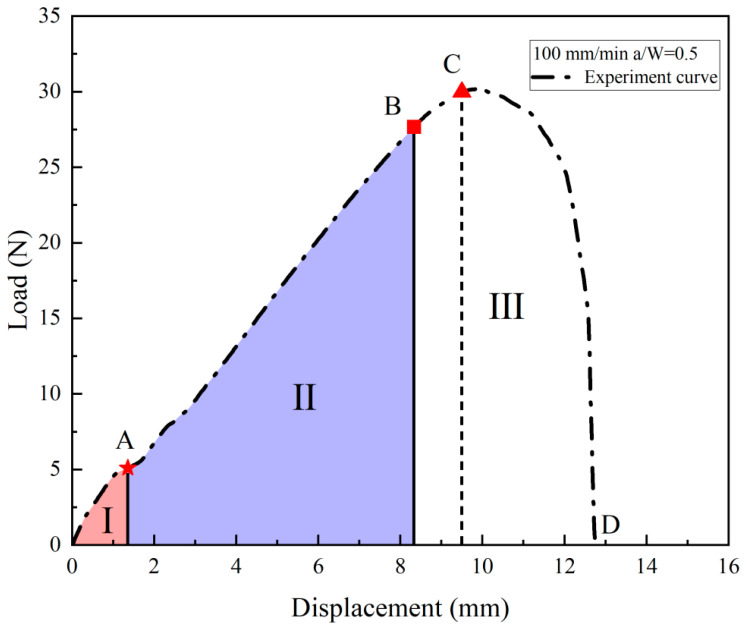
Displacement–load curve for a specimen under 100 mm/min tensile rates.

**Figure 12:**
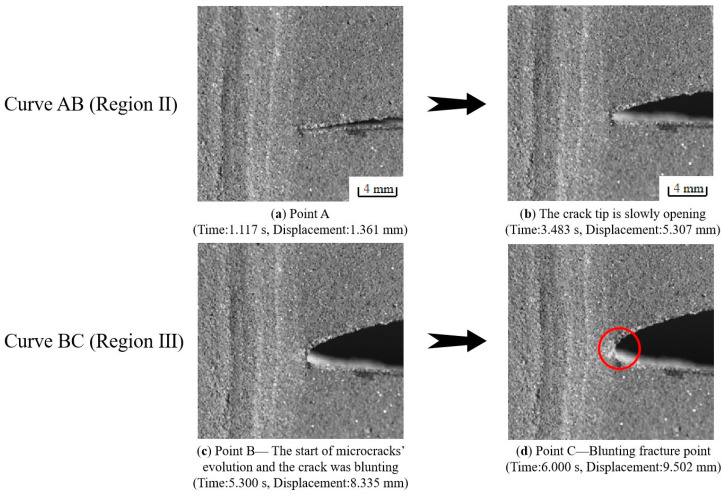

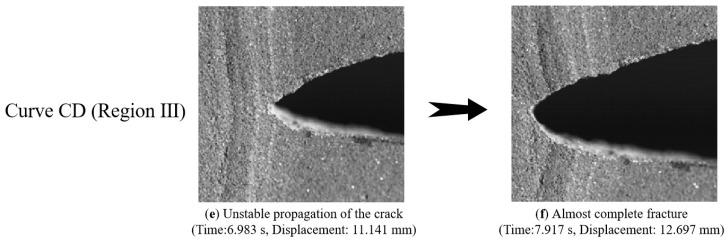
Crack tip behavior in the whole process of crack propagation corresponding to Curve AD in Figure 11.

As shown in Figure 11, regions II and III of the load–displacement curve were separated by four nodes, ABCD, and the crack tip behavior characteristics were studied for each curve. Curve AB in Figure 11 corresponds to Figure 12a,b, which represents the initial stage of stretching with minimal crack propagation. Then the NEPE propellant material entered the process of “damage evolution and crack propagation”, which corresponded to Figure 12c,d and the BC curve in Figure 11. At this stage, the elastic stiffness of the material began to decline. In contrast to the AB section of the curve, the crack opening displacement in this region increases significantly, but the crack tip’s propagation distance in the x-direction remains short. Therefore, the energy input into the system through the tensile load is primarily dissipated by the deformation of the propellant material. Additionally, the crack tip of the NEPE propellant exhibits significant blunting at this stage. 

**Figure 13:**
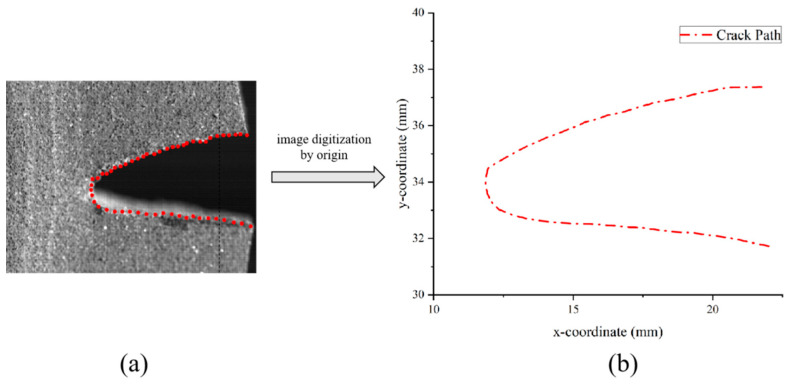
Blunting fracture of crack tip (point C in Figure 14): (**a**) feature point selection by origin corresponding to Figure 12d; (**b**) output of the feature point coordinates in origin.

**Figure 14:**
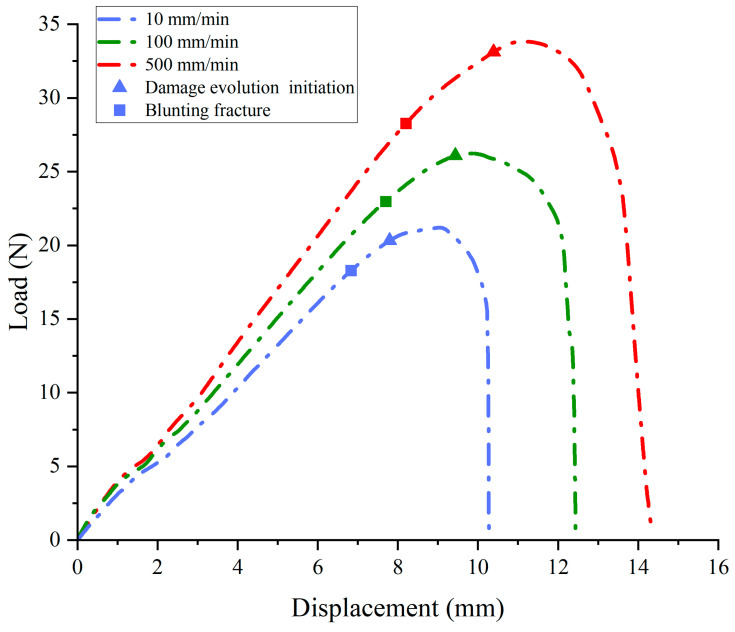
Average load–displacement curves under different loading rates.

Figure 13 shows the image digital processing of the crack path when the NEPE propellant is positioned at point C on the curve using origin software. At this time, the crack tip is obviously blunted, and point C represents that the crack tip has reached the blunting limit, which is referred to as the blunting fracture point [41]. The load corresponding to the blunting fracture point is designated as the critical load. The study of blunting fracture by scholars indicates that prior to the attainment of the critical load, the crack tip is only blunted, and the crack cannot be propagated while the fracture cannot be triggered [42]. This is consistent with the results observed in this experiment. Figure 14 illustrates the average load–displacement curves under different loading rates. It can be observed that an increase in loading rate leads to a higher critical load. The critical loads of the NEPE propellant tests under the three loading rates are 20.326 N, 26.085 N, and 33.114 N, respectively. It is well established that NEPE propellants are subject to a variety of loads over their operational lifetime, and cracks can develop in propellants. However, the development of cracks in the propellant does not necessarily indicate the end of its useful life. It is possible to extend the service life of the NEPE propellant by controlling the load to below the ultimate load.

When the propellant fracture process enters the CD curve corresponding to Figure 11, more obvious crack propagation and fracture processes will occur. At this stage, the NEPE propellant is in the “rapid rupture zone”, and its stiffness decreases significantly due to the rapid crack propagation. Under external load, cracks rapidly spread in the final stage until the propellant material finally fails completely.

**Figure 15:**
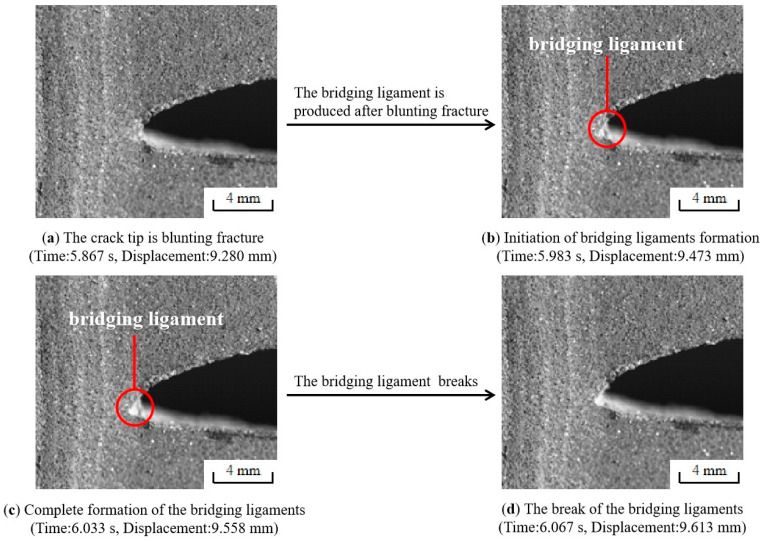
Formation and break of the bridging ligaments during blunting fracture.

**Figure 16:**
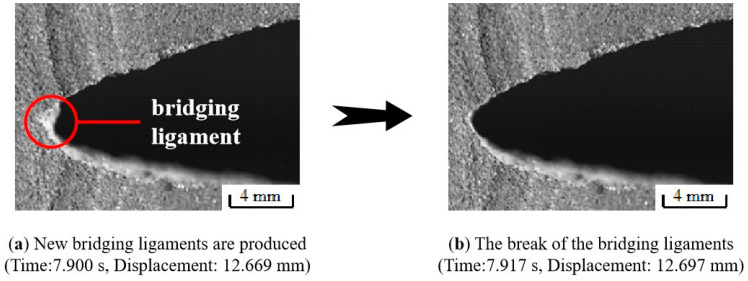
The generation and destruction of bridging ligaments throughout the whole process of crack propagation.

The behavior of the crack tip during the fracture process of the NEPE propellant specimen was recorded by the high-speed camera system and then analyzed. As shown in Figure 15, a large number of bridging ligaments will be formed at the crack tip after blunting fracture in region III. Subsequently, the bridge ligaments break, causing micro-cracks and micro-voids to merge with the main cracks, resulting in macroscopic crack propagation in the propellant. However, new bridging ligaments will be generated at the same time of fracture. When the propellant is completely broken, there will be no remaining bridging ligament at the crack tip. This means that the generation and destruction of bridging ligaments occur throughout the entire process of crack propagation, as shown in Figure 16. The above results indicate that the formation of bridging ligaments is a prerequisite for macroscopic crack propagation. The fracture process in the NEPE propellant involves alternating blunting fracture and crack propagation at the crack tip. In other words, crack propagation and suppression exist simultaneously. As the bridge ligaments always form before breaking, the blunting of the crack always precedes propagation. Therefore, the propagation of the macroscopic crack is not a completely continuous process, with the instantaneous speed of crack propagation also fluctuating. Similarly, Wang’s research demonstrated that crack growth in the HTPB propellant also occurs through a blunt–growth–blunt–growth mechanism of extension, which is highly nonlinear [13].

**Figure 17:**
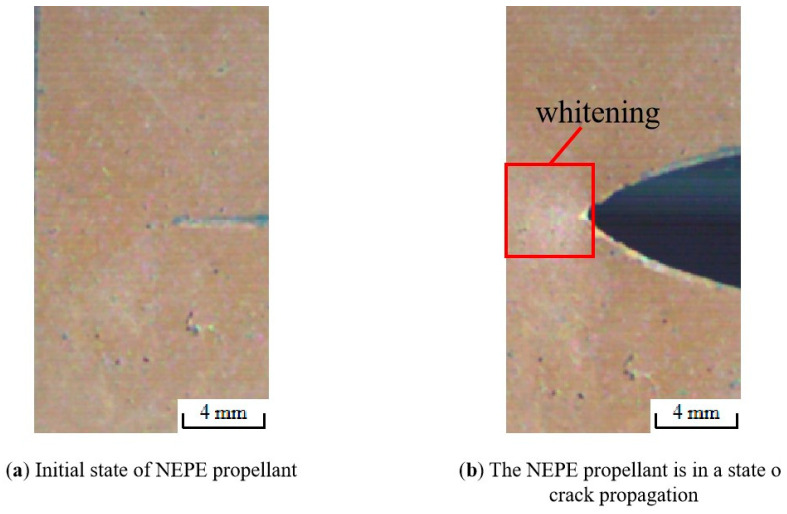
Dewetting damage of NEPE propellant during the crack propagation.

Additionally, during crack propagation, a “whitening” phenomenon and small crystal precipitation were observed near the crack tip. High-speed camera color images of the NEPE propellant in Figure 17 show that the crack tip of the specimen became noticeably “white” near the root when the propellant stretching process entered region III. During the stretching process, the “white” area would increase, which indicates that the material will cause dewetting damage during crack propagation. The modulus of solid fillers AP/Al of NEPE propellants is considerably greater than that of the binder. While the addition of solid fillers can enhance the overall strength of the propellant material, the disparity in the modulus of this component gives rise to a localized stress concentration between the interface of large particle size and the base material. This results in microcrack initiation or particle/binder separation (dewetting). In other words, during the crack propagation in NEPE propellants, damage occurs in two forms: one in the form of micro-cracks and micro-holes and the other in the form of dewetting. Liu CT’s theory demonstrates that these damage initiation and evolution processes are time-dependent, resulting in material strength degradation and fracture behavior that are time-sensitive [43].

**Figure 18:**
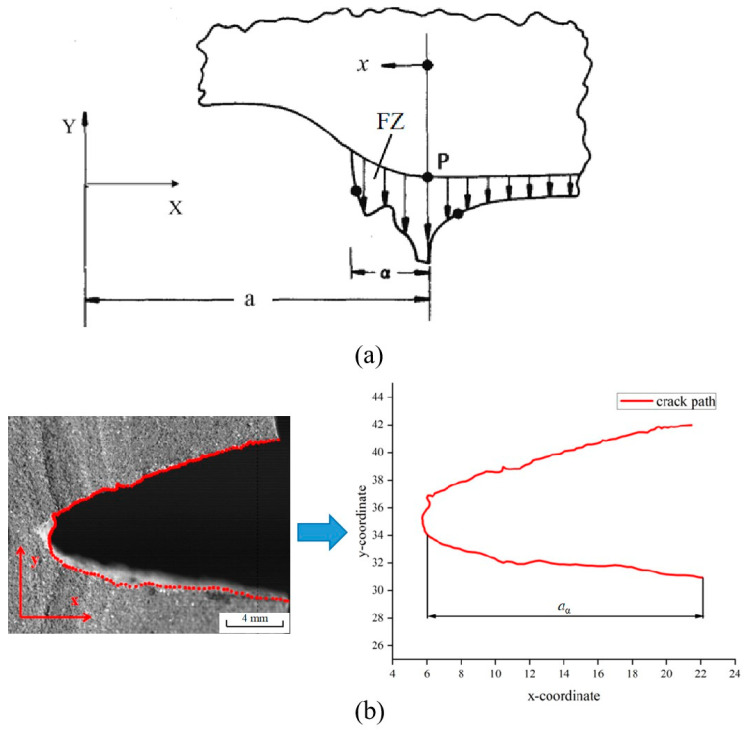
Schematic diagram of “apparent crack tip”: (**a**) crack tip P and the failure zone [45]; (**b**) method of obtaining the length of “apparent crack tip”.

The determination of the crack propagation velocity necessitates the analysis of discrete data pertaining to the instantaneous time *t* and the corresponding length a. Schapery’s theory suggests that in practical application, the crack propagation velocity of the propellant can be determined by measuring the increase in the length of the “apparent crack tip”: over a unit of time [44]. The crack tip is situated within the failure zone (FZ). This zone is where the continuum material begins to come apart and eventually separates entirely, and its dimension α can not be measured accurately. Consequently, the “apparent crack length” can be expressed as the actual crack length minus the length of the failure zone: *a_α_* = *a* − *α* [45]. The “apparent crack tip” length *a_α_* is defined in Figure 18, and the equation for crack propagation velocity is given by:

(13) $$V=\frac{d(a-\alpha )}{dt}=\frac{da_{\propto }}{dt}$$

The process of crack evolution in NEPE propellant can be captured by high-speed cameras, allowing for the measurement of the “apparent crack tip” length at different times through digital imaging. Firstly, the image is divided into 50 equal sections, after which the image is selected at equal intervals. The total number of frames in each section is then divided by the set frame rate to determine the corresponding moment of the image. As the study concerns the crack propagation rate in the x-direction, it is only necessary to obtain the x-coordinate of the crack tip and the crack initiation position to determine the length of the “apparent crack tip”. Subsequently, the apparent crack length was recorded at fixed time intervals, and the increase in crack length in the x-direction at fixed time intervals was calculated in order to obtain the crack propagation rate law.

**Figure 19:**
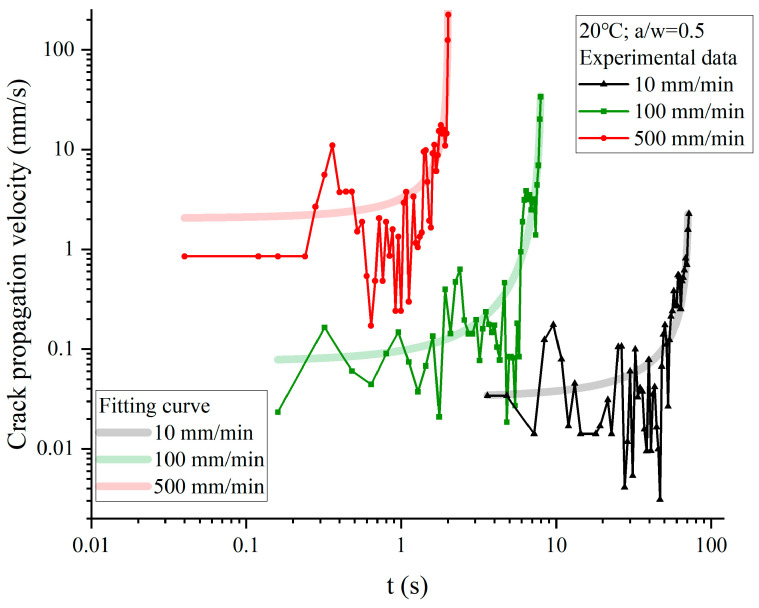
The relationship between crack propagation velocity along the x-direction and time under different tensile rates: experiment curves and fitting curves.

The curves of crack propagation velocity along the x-direction and time under different tensile rates are shown in Figure 19. The results indicate that the crack propagation velocity of NEPE propellant increases exponentially with stretching. At the beginning of tensile fracture, the crack of NEPE propellant opens slowly, and the crack propagation velocity is almost zero. Subsequently, when the crack began to propagate, the velocity along the x-direction increased rapidly. However, it should be noted that crack propagation velocity is not a monotonically increasing process. The study mentioned above found that the crack tip blunting and propagation during the crack evolution of the NEPE propellant alternate, indicating that the velocity law of crack propagation is consistent with experimental observations. When the crack tip blunts, the process of crack propagation will be weakened, causing a decrease in velocity along the x-direction. The results of the crack propagation velocity also show that the blunting and propagation almost always run through the whole fracture process of the NEPE propellant, but with the increase in the stretching rate, the blunting fracture will gradually weaken. Therefore, if the stretching rate is sufficiently high, it is hypothesized that the blunting process will not occur during the crack propagation of the NEPE propellant.

The aforementioned phenomenon indicates that blunting fracture occurs during the entire NEPE propellant fracture process, and the following conclusions can be drawn: (1) Prior to the macroscopic propagation of cracks, the blunting process exerts a dominant influence on the fracture process. In the event that the load on NEPE propellant does not reach the critical load, the crack is only blunted, rather than propagating. Furthermore, the limit load increases with the increase in the loading rate. (2) The formation of bridging ligaments is a prerequisite for macroscopic crack propagation as the bridge ligaments always form before breaking, and the blunting of the crack always precedes propagation. (3) During the crack propagation in NEPE propellants, damage occurs in two forms: one in the form of micro-cracks and micro-holes and the other in the form of dewetting. (4) The velocity curve of crack growth exhibits fluctuations due to the continuous blunting of the NEPE propellant during the process of crack propagation. When blunting fracture occurs, the crack tip rate decreases. The entire process of crack propagation can be described as a series of blunt–propagate–blunt–propagate cycles.

#### 4.1.3. Fracture Toughness Analysis

The energy release rate, which represents the energy required per unit area of propellant crack propagation, is an important parameter in the numerical simulation of peridynamics. Many previous studies have demonstrated that the stress intensity factor *K* can be used to describe the fracture toughness of propellant materials, and it represents the strength of the load and deformation at the crack tip [46,47]. Therefore, the critical stress intensity factor *K_I_*_c_ and energy release rate *G* are calculated to analyze the fracture toughness of the NEPE propellant. 

As the size of the test specimen is small, the dead weight of the test specimen has little influence on the energy release rate. The simplified Formula (14) can be used to obtain the energy release rate of the NEPE propellant [48]:

(14) G=U/(B(W−a))

where *U* is the elastic strain energy, a is the length of the initial crack, and *W* represents the width of the test specimen. Table 3 shows *G* for different loading rates. When the loading rate increases, the energy release rate increases.

In fracture mechanics, the critical stress intensity factor *K_I_*_c_ can be expressed as [49]

(15) $$K_{Ic}=\sigma_{B}\sqrt{\pi a}\sqrt{\frac{2W}{\pi a}tan\frac{\pi a}{2W}}G_{I}(a/W)$$

(16) $$G_{I}=\frac{0.752+2.02\frac{a}{W}+{0.37(1-sin\frac{\pi a}{2W})}^{3}}{cos\frac{\pi a}{2W}}$$

where $\sigma_{B}$ is the stress corresponding to the blunting fracture point. It is crucial to emphasize that $G_{I}$ represents a size-dependent coefficient, rather than the energy release rate.

**Figure 20:**
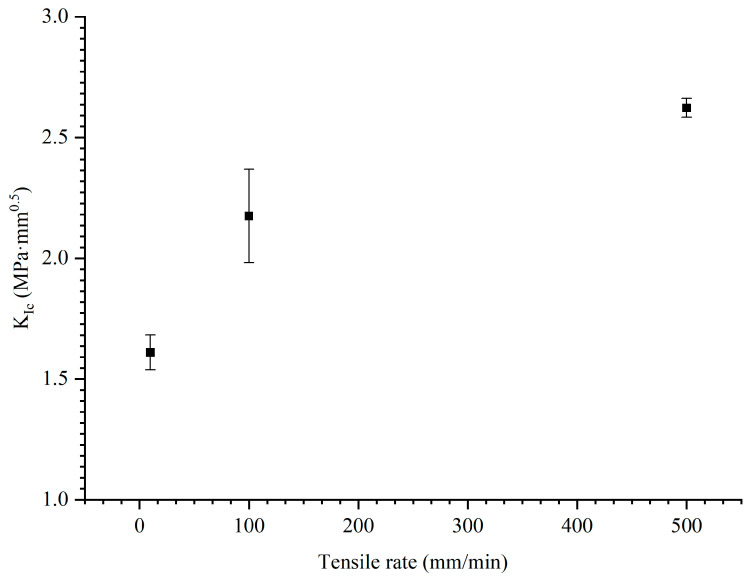
The critical stress intensity factor *K_Ic_* vs. Loading rates.

The relationship between the value of stress intensity factor *K_Ic_* and the loading rate is shown in Figure 20. As the loading rate is increased, the *K_Ic_* of the NEPE propellant material also increases. This implies that a large loading rate will enhance the fracture toughness of the propellant. However, as the loading rate continues to increase, the growth rate of fracture toughness slows down, which indicates that there is a limit to the effect of loading rate on the fracture toughness of NEPE propellant. The fracture toughness of NEPE propellants is rate-dependent and may eventually become constant as the loading rate increases, but this requires further research.

The statistical analysis indicates that 98.4% of solid rocket motor ignition failures are attributable to the propagation of propellant cracks [6], but the presence of cracks does not mean that NEPE propellants are entirely unusable. The above research results provide a potential criterion for the use of NEPE propellants: When the maximum stress intensity factor *K*_max_ before the complete combustion of both ends of the material crack is less than the critical stress intensity factor *K_Ic_* (*K*_max_ ≤ *K_Ic_*), the propellant does not exhibit crack propagation during operation, and the SRM can normally ignite. The burning rate and ignition pressure of SRM are known design parameters. It is convenient to obtain the crack condition of the NEPE propellant using micro-computed tomography, synchrotron radiation light sources, or other technologies. The change in the stress intensity factor before complete burnout at both ends of the crack under the stress state of combustion can be studied in experiments. If the stress intensity factor is less than the critical value during this period, SRM can be used as normal. Otherwise, the SRM should be scrapped. 

### 4.2. Numerical Calculation of BBPD

**Figure 21:**
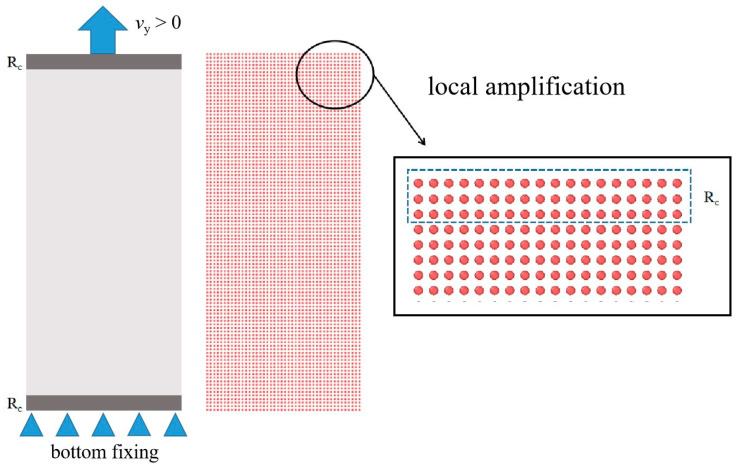
Boundary region and material points model.

Considering Figure 21, the specimen is modeled with 4961 material points with a spacing of 0.5 mm. The internal length parameter is δ=3.134∆x.

**Figure 22:**
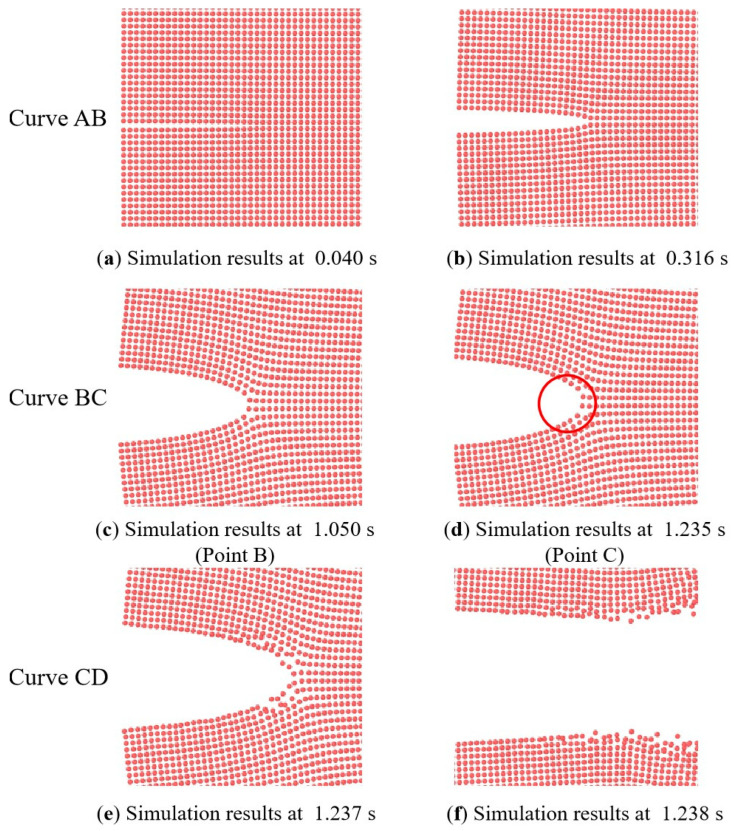
The BBPD model (Local area): calculation results for 500 mm/min tensile rate.

**Figure 23:**
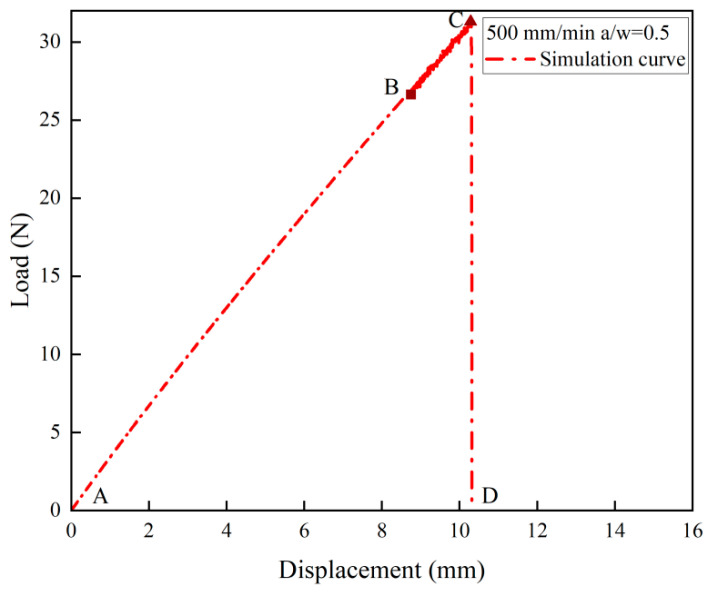
Simulation load–displacement curve under 500 mm/min tensile rate.

**Figure 24:**
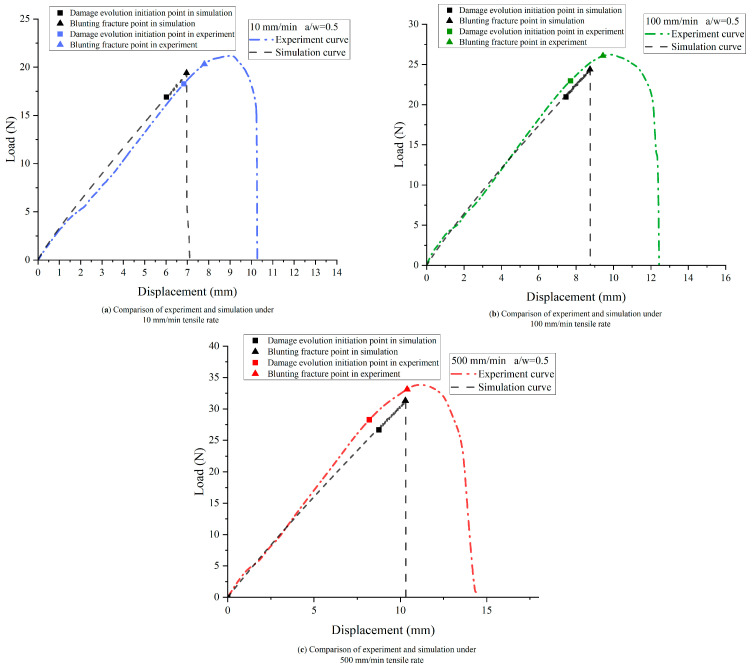
Load–displacement curves of experiment and simulation under different mm/min tensile rates.

The load–displacement curve and crack propagation velocity of the NEPE propellant for different loading rates can be obtained from the simulation. The velocity constraint is applied to the upper bound of the SENT specimen at three different rates, corresponding to the experiment. Figure 22 presents close-up views of the crack tip, demonstrating that BBPD is an effective method for capturing the blunting process of NEPE propellant. The simulation results align with the experimental observations obtained through high-speed photography, providing confidence in the accuracy of the simulation. Figure 23 and Figure 24 compare the load–deflection curves of the simulation and experiments. The damage nucleation at point B and blunting fracture at point C are consistent with the experiment observations. However, in the blunting fracture stage, the simulation curve exhibits an unstable fluctuation reflecting the blunting fracture process of the NEPE propellant. Once the fracture process of NEPE propellant reaches the blunting fracture point C, although crack propagation occurs, the strength of the material does not decrease accordingly, and this leads to faster crack propagation times in simulations compared to experiments. The improvement of the constitutive model and the transition to methodologies such as the nonlocal operator method may potentially resolve these issues, which will be the subject of future research. Nevertheless, the simulation of load–displacement curves still reflects the fracture characteristics of the NEPE propellant during crack propagation, which indicates that the BBPD method is effective in predicting the fracture process of the NEPE propellant.

**Figure 25:**
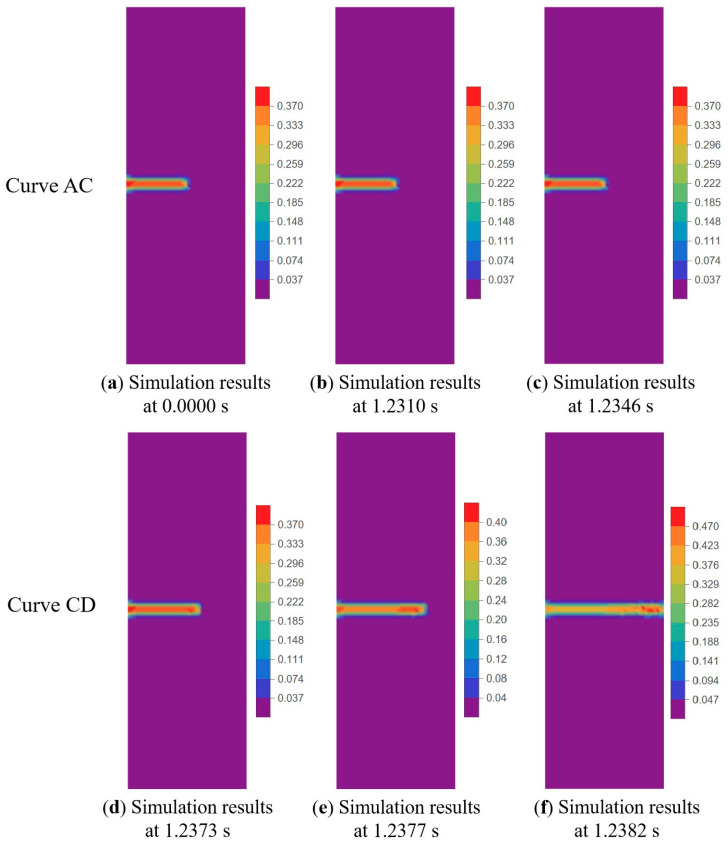
Phase field diagram (damage nephogram) of crack during tensile process (500 mm/min tensile rate).

The output result of BBPD can be visualized to obtain the crack phase-field diagram, which allows for a visual description of the change in damage degree of each part of the propellant material during the tensile fracture process. As shown in Figure 25, when the NEPE propellant is within the linear viscoelastic region (Curve AB in Figure 23), the damage nephogram demonstrates minimal change, indicating that the NEPE propellant does not exhibit any significant mechanical damage behavior prior to crack propagation. Upon entering the “damage evolution and crack propagation” region (Curve BD in Figure 23), a typical damage phenomenon is observed within the NEPE propellant. This result is consistent with the experimental study presented in the literature [8].

**Figure 26:**
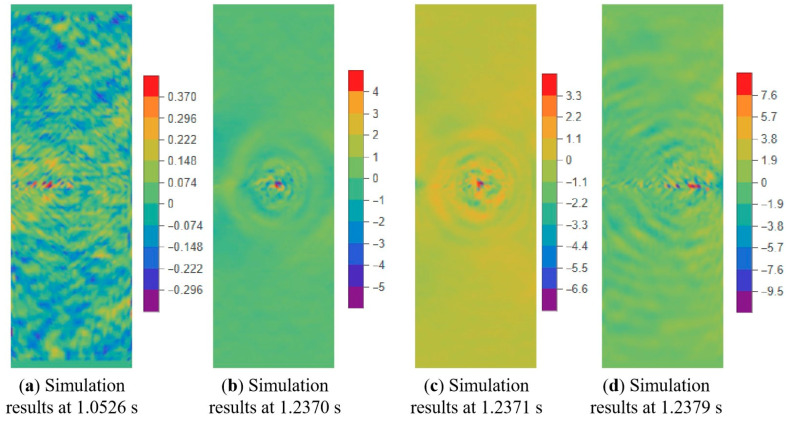
Velocity nephogram (x-direction) of crack during tensile process (500 mm/min tensile rate).

**Figure 27:**
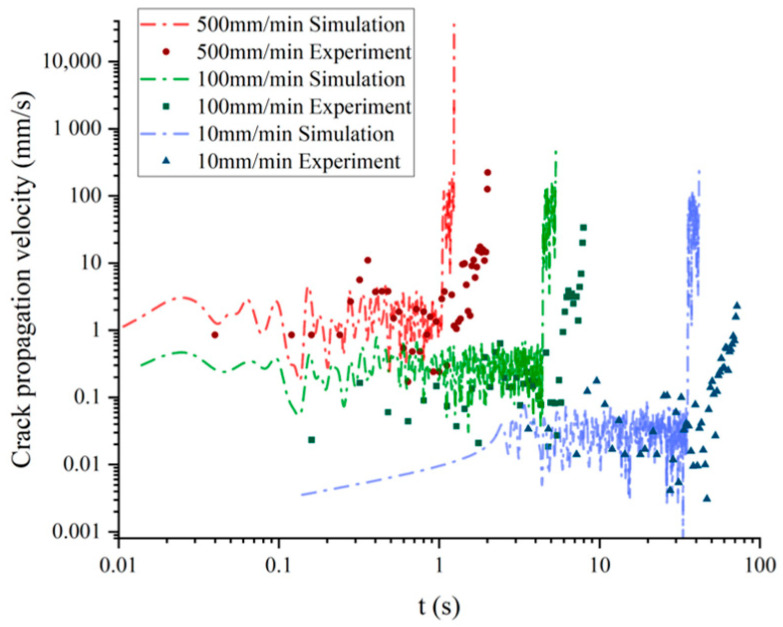
Comparison of experiments and simulations under 500 mm/min for crack propagation velocity.

In the tensile experiment, the crack speed can only be calculated through frame analysis of photos taken by a high-speed camera. Figure 26 suggests that the crack speed in the x-direction is almost zero before crack propagation occurs. Once the crack propagates, the velocity of the crack tip increases rapidly. Figure 27 compares the crack tip speed of the simulation with the experiments. Consistent with the experiment, the increase in crack propagation velocity is not a completely monotonic process in the simulation. The simulation results indicate that using BBPD can predict crack propagation velocity well.

## 5. Conclusions

In this study, the fracture behavior and characteristics of nitrate ester-plasticized polyether propellants have been studied in detail. The mechanical response, crack morphology, and evolution during crack propagation were studied using the combination of a drawing machine and a high-speed camera. The stress intensity factor and energy release rate of the material were calculated through experiments and used for numerical simulation. The load–displacement curves and crack propagation velocity at different tensile rates were measured in experiments, and BBPD was used to simulate and predict the crack propagation of NEPE propellant for the first time. The main conclusions are as follows:

1. The load–displacement curve obtained by the experiment can be divided into three regions. At the beginning, the contact surface between the chuck of the stretching machine and the specimen reaches an ideal state and the crack opens slowly. Then, the propellant undergoes blunting fracture and unstable propagation. During blunting fracture, a large number of bridging ligaments form at the crack tip, and the crack growth rate is minimal. Finally, the crack propagation velocity increases exponentially.
2. The crack propagation velocity along the x-direction of the NEPE propellant increased with the increasing loading rate and crack blunting was observed, resulting in fluctuating crack propagation velocity. The entire process of crack propagation in NEPE propellants can be described as a series of blunt–propagate–blunt–propagate cycles.
3. Both the energy release rate and the stress intensity factor are affected by the rate-dependent characteristics of the NEPE propellant. The critical stress intensity factor *K_Ic_* and the energy release rate G both increase with an increasing loading rate. A criterion related to the stress intensity factor is proposed as a means of determining whether SRM can normally ignite.
4. Bond-based peridynamics (BBPD) can simulate the fracture process of the NEPE propellant well. It obtains the velocity field, damage field (crack phase field), and load–displacement curve of the NEPE propellant. In future work, the fracture characteristics of NEPE propellants at different temperatures and confining pressures can be considered. It is also necessary to improve the simulation accuracy by using a nonlinear viscoelastic constitutive model with higher precision and develop an associated peridynamic model.

## Figures and Tables

**Table 1:** Formation of the investigated NEPE propellant.

Binder/%	Plasticiser/%	AP/%	Al/%	HMX/%	Additives/%
6–8	17–21	20–25	10–12	40–45	1–3

**Table 2:** Parameters in the Prony series for relaxation modulus of solid propellant [31].

n	∞	1	2	3	4	5
$\tau_{n}^{E}$/s	—	0.5	2	32	128	512
*E_n_*/MPa	2.786	0.362	0.718	0.336	0.548	0.530

**Table 3:** The value of *G*.

	10 mm/min	100 mm/min	500 mm/min
*G* (J/m^2^)	1279.3080	2412.1082	3851.1036

## Data Availability

The data that support the findings of this study are available from the corresponding author [Xiaolong Fu] upon reasonable request.

## Nomenclature

Nomenclature		$\Delta t_{cr}$	critical time step
*a*	crack length	∆x	distance between two adjacent points
*B*	thick of the specimen	α	length of the failure zone
*W*	wide of the specimen	*a_α_*	apparent crack length
*H*	height of the specimen	*K* *_I_*_c_	critical stress intensity factor for mode I
$E_{\infty }$	long-term equilibrium modulus	*K*	stress intensity factor
$N_{E}$	number of terms of Prony series		
*E*_0_	initial stress relaxation modulus	Abbreviation	
*R*	material region		
*x*	material point	SRM	solid rocket motors
δ	horizon size of material point *x*	NEPE	nitrate ester plasticized polyether
*H_x_*	family of material point x	AP	ammonium perchlorate
ρ	density	HMX	octogen
*u*	displacement	CMDB	composite modified doublebase
*b*	body force density	HTPB	hydroxyl-terminated polybutadiene
f	peridynamic force density	FEM	finite element method
ξ	position vector	XFEM	extended finite element method
η	displacement vector	CZM	cohesive zone model
c	bond constant	PD	peridynamic
*s*	bond stretch	PBX	polymeric-bonded explosive
*G_c_*	critical energy release rate	BBPD	bond-based peridynamic
μ	damage function	SENT	single-edge notched tension
φ	damage parameter	FZ	failure zone

## References

[1] Wang Y., Rong H., Zhang X., Chen Y., Luo W., Liu Y., Yao W., Tian Y. (2021). Influences of Bu-NENA and BDNPA/F Plasticizers on the Properties of Binder for High-Energy NEPE Propellants. Propellants Explo. Pyrotec..

[2] Yan G., Liu W., Wang Q., Du J., Wang F., Yu S. (2019). Investigation Aging Behavior of High Energy Propellant Materials. IOP Conf. Ser. Mater. Sci. Eng..

[3] Yan X., Xia Z., Huang L., Ma L., Na X., Feng Y., Fang C. (2020). Study on the Ignition Process and Characteristics of the Nitrate Ester Plasticized Polyether Propellant. Int. J. Aerosp. Eng..

[4] Hu Q., Fang Q., Sha B., Jin L. (2022). Study on the Viscoelastic Damage Properties of NEPE Solid Propellant with Different Cyclic Stress Ratios. Propellants Explo. Pyrotec..

[5] Xing R., Wang L., Zhang F., Hou C. (2022). Mechanical Behavior and Constitutive Model of NEPE Solid Propellant in Finite Deformation. Mech. Mater..

[6] Jiang Q., Lv X., Cui H., Ma T. (2023). Computational Technique for Crack Propagation Simulation in Viscoelastic Solid Propellant. Int. J. Aerosp. Eng..

[7] Han B., Ju Y., Zhou C. (2012). Simulation of crack propagation in HTPB propellant using cohesive zone model. Eng. Fail. Anal..

[8] Li M. (2022). Mechanical Behaviors and Constitutive Relations under Wide Strain Rate Range for CMDB Propellant. Polym. Test..

[9] Zhang H., Liu M., Miao Y., Wang H., Chen T., Fan X., Chang H. (2020). Dynamic Mechanical Response and Damage Mechanism of HTPB Propellant under Impact Loading. Materials.

[10] Qiang H., Wang J., Wang Z., Wang X., Zhu Z. (2023). Research progress on strength, damage and fracture failure of composite solid propellants. Chin. J. Explos. Propellants.

[11] Wang Z., Qiang H., Wang J., Duan L. (2022). Experimental Investigation on Fracture Properties of HTPB Propellant with Circumferentially Notched Cylinder Sample. Propellants Explo. Pyrotec..

[12] Wang W., Zheng J., Xu J., Chen X., Zhou C. (2015). Research on fracture mechanism of AP/Al/CMDB propellant. J. Propuls. Technol..

[13] Wang T. (2021). Crack Propagation Velocity and Fracture Toughness of Hydroxyl-Terminated Polybutadiene Propellants: Experiments and Simulations. Eng. Fract. Mech..

[14] Ghabezi P., Farahani M. (2017). Effects of Nanoparticles on Nanocomposites Mode I and II Fracture: A Critical Review. Rev. Adhes. Adhes..

[15] Ghabezi P., Farahani M. (2018). Characterization of Cohesive Model and Bridging Laws in Mode I and II Fracture in Nano Composite Laminates. JMES.

[16] Wubuliaisan M., Wu Y., Hou X., Duan H., Huang F. (2023). Viscoelastic Debonding Criterion-Based Interface for Modeling the Mechanical Behavior of Solid Propellants Subjected to Large Deformation. Eur. J. Mech..

[17] Cui H. (2022). Viscoelastic Cohesive Zone Modeling for Mode II Fracture between Propellant and Insulation. Int. J. Adhes. Adhes..

[18] Gao B., Li Z., Ji Y. (2022). Experimental and XFEM Simulation Research on HTPB Propellant Precracks Based on Microscopic Observation and DIC. Propellants Explo. Pyrotec..

[19] Cui H. (2022). Numerical Simulation of Crack Propagation in Solid Propellant with Extrinsic Cohesive Zone Model. Meccanica.

[20] Mandenci E., Oterkus E. (2014). Peridynamic Theory and Its Applications.

[21] Silling S.A. (2000). Reformulation of elasticity theory for discontinuities and long-range forces. J. Mech. Phys. Solids.

[22] Silling S.A., Askari E. (2005). A meshfree method based on the peridynamic model of solid mechanics. Comput. Struct..

[23] Deng X., Zhao J., Huang Y. (2023). Peridynamic Modeling of Damage and Non-Shock Ignition Behavior of Confined Polymer Bonded Explosives under Impact Loading. Phys. Scr..

[24] Ghajari M., Iannucci L., Curtis P. (2014). A Peridynamic Material Model for the Analysis of Dynamic Crack Propagation in Orthotropic Media. Comput. Methods Appl. Mech. Eng..

[25] Sun W.K., Yin B.B., Sun J., Kodur V.K.R., Liew K.M. (2024). Modeling via Peridynamics for Crack Propagation in Laminated Glass under Fire. Compos. Struct..

[26] Kumar S. (2024). Modelling Ductile Damage in Metals and Alloys through Weyl Condition Exploiting Local Gauge Symmetries. Int. J. Solids Struct..

[27] Isiet M., Mišković I., Mišković S. (2021). Review of Peridynamic Modelling of Material Failure and Damage Due to Impact. Int. J. Impact Eng..

[28] Deng X.L., Wang B. (2020). Peridynamic modeling of dynamic damage of polymer bonded explosive. Comput. Mater. Sci..

[29] Bentil S.A., Jackson W.J., Williams C., Miller T.C. (2022). Viscoelastic Properties of Inert Solid Rocket Propellants Exposed to a Shock Wave. Propellants Explo. Pyrotec..

[30] Luo R. (2018). Development of Prony Series Models Based on Continuous Relaxation Spectrums for Relaxation Moduli Determined Using Creep Tests. Constr. Build. Mater..

[31] Hu S. (2015). A Visco-Hyperelastic Constitutive Model for NEPE Propellant and Its Application.

[32] Safari-Naderi M.-H., Shakouri M., Ghasemi-Ghalebahman A. (2023). A Bond-Based Peridynamics Model Based on Variable Material Properties for Modeling Elastoplastic Behavior. Mater. Today Commun..

[33] Dimola N., Coclite A., Fanizza G., Politi T. (2022). Bond-Based Peridynamics, a Survey Prospecting Nonlocal Theories of Fluid-Dynamics. Adv. Cont. Discr. Mod..

[34] Sun M., Liu L., Mei H., Lai X., Liu X., Zhang J. (2023). A Bond-Based Peridynamic Model with Matrix Plasticity for Impact Damage Analysis of Composite Materials. Materials.

[35] Ladányi G., Gonda V. (2021). Review of Peridynamics: Theory, Applications, and Future Perspectives. SV-JME.

[36] Dipasquale D., Sarego G., Zaccariotto M., Galvanetto U. (2016). Dependence of Crack Paths on the Orientation of Regular 2D Peridynamic Grids. Eng. Fract. Mech..

[37] Spreiter Q., Walter M. (1999). Classical molecular dynamics simulation with the Velocity Verlet algorithm at strong external magnetic fields. J. Comput. Phys..

[38] Wang T., Xu J., Li H., Chen X., Zhang J. (2023). Crack Propagation Velocity and Fracture Toughness of Hydroxyl-Terminated Polybutadiene Propellants with Consideration of a Thermo-Viscoelastic Constitutive Model: Experimental and Numerical Study. Theor. Appl. Fract. Mech..

[39] Zhang H., Chang H., Li J., Li X., Wang H. (2021). High-Strain-Rate Mechanical Response of HTPE Propellant under SHPB Impact Loading. AIP Adv..

[40] Zhang H., Chang H., Li X., Wu X., He Q. (2022). The Effect of Strain Rate on Compressive Behavior and Failure Mechanism of CMDB Propellant. Def. Technol..

[41] Chen J.H., Wang Q., Wang G.Z., Li Z. (2003). Fracture Behavior at Crack Tip—A New Framework for Cleavage Mechanism of Steel. Acta Mater..

[42] Doungkaew N., Eichhubl P. (2024). Fracture Ellipticity as a Measure of Chemical Reaction-Controlled Fracture Growth. J. Struct. Geol..

[43] Liu C.T., Laboratory P. (1997). Crack growth behavior in a solid propellant. Eng. Fract. Mech..

[44] Schapery R.A. (1975). A theory of crack initiation and growth in viscoelastic media I. Theoretical development. Int. J. Fract..

[45] Schapery R.A. (2022). A Theory of Viscoelastic Crack Growth: Revisited. Int. J. Fract..

[46] Hou X., Fan L., Yu C., Zhong J. (2021). Crack Propagation Analysis of Damaged Solid Propellant under Impact Overload. J. Phys. Conf. Ser..

[47] Martínez M., López R., Rodríguez J., Salazar A. (2022). Evaluation of the Structural Integrity of Solid Rocket Propellant by Means of the Viscoelastic Fracture Mechanics Approach at Low and Medium Strain Rates. Theor. Appl. Fract. Mech..

[48] Gao D., Zhang T. (2007). Fracture energy of steel fiber reinforced high strength concrete under three-point bending. Shuili Xuebao.

[49] Gross D., Seelig T. (2018). Linear Fracture Mechanics. Fracture Mechanics.

